# A Novel Electrochemical Differentiation between Exosomal-RNA of Breast Cancer MCF7 and MCF7/ADR-Resistant Cells

**DOI:** 10.3390/ph16040540

**Published:** 2023-04-04

**Authors:** Mohammed H. Abdelaziz, Ehab N. El Sawy, Anwar Abdelnaser

**Affiliations:** 1 Chemistry Department, School of Sciences and Engineering, The American University in Cairo, New Cairo 11835, Egypt; 2 Institute of Global Health and Human Ecology, The American University in Cairo, New Cairo 11835, Egypt

**Keywords:** cancer, cancer resistance, cancer diagnosis, cancer classification, extracellular vesicles (EVs), biomarker, assay, exosomes, MCF7, cell culture, MCF7/ADR, SH-SY5Y, HEPG2, exosomes isolation, exosomal RNA, exosomes characterization, cyclic voltammetry (CV), square wave voltammetry (SWV), differential pulse voltammetry (DPV), normal pulse voltammetry (NPV), scanning electron microscope (SEM), transmission electron microscope (TEM), zeta-sizer, zeta potential, screen-printed electrodes (SPE), gold electrode, peak current suppression, peak current shifting, adsorption

## Abstract

Cancer is considered one of the most burdensome diseases affecting lives and, hence, the economy. Breast cancer is one of the most common types of cancer. Patients with breast cancer are divided into two groups: one group responds to the chemotherapy, and the other group resists the chemotherapy. Unfortunately, the group which resists the chemotherapy is still suffering the pain associated with the severe side effects of the chemotherapy. Therefore, there is a critical need for a method to differentiate between both groups before the administration of the chemotherapy. Exosomes, the recently discovered nano-vesicles, are often used as cancer diagnostic biomarkers as their unique composition allows them to represent their parental cells, which makes them promising indicators for tumor prognosis. Exosomes contain proteins, lipids, and RNA that exist in most body fluids and are expelled by multiple cell types, including cancer cells. Furthermore, exosomal RNA has been significantly used as a promising biomarker for tumor prognosis. Herein, we have developed an electrochemical system that could successfully differentiate between MCF7 and MCF7/ADR depending on the exosomal RNA. The high sensitivity of the proposed electrochemical assay opens the door for further investigation that will address the other type of cancer cells.

## 1. Introduction

According to the World Health Organization (WHO), the number of new cancer patients has dramatically exceeded 18 million patients per year, and the number of cancer deaths has reached around 10 million [1]. On an economic scale, the annual cost of cancer is over USD 1.5 trillion [2]. An early cancer diagnosis can significantly increase the survival percentage to above 90% from 26%, which is the survival percentage for late-stage diagnosis; therefore, early cancer diagnosis is critical for cancer treatment [3].

The need to develop electrochemical and pain-free methods with which to accurately diagnose cancer has significantly increased with the booming number of cancer patients and the pain associated with biopsy for cancer diagnosis. Exosomes can be used as potential biomarkers for cancer diagnosis [4]. In this work, we report simple assays to differentiate between MCF7, MCF7/ADR, SH-5YSY, and HepG2 cells. Zeta potential as a characteristic property of the exosomes population was used to differentiate between the exosomes of the MCF7, SH-5YSY, and HepG2 cells.

Breast cancer is one of the most common types of cancer, especially in women [5]. The most common chemotherapy for breast cancer is Adriamycin, while resistance development and side effects of the long-term administration of adriamycin are the chief challenges of using it as chemotherapy in all cancer cases [6]. The phenomenon of cancer cells being resistant to anticancer drugs is called multidrug resistance (MDR), and MDR can be considered one of the main obstacles to cancer treatment [7].

The causes of MDR were extensively studied during the last three decades and assigned to numerous mechanisms. For instance, the active efflux of anticancer drugs by a specific type of pump called a drug efflux pump is one of the chief mechanisms of developing MDR [7]. Another contributor to chemotherapy resistance is the ATP-binding cassettes (ABC). ABC transporters are bacterial-membraned proteins and their major function is substrate efflux [7]. These types of transporters are located in the cell membrane or the membranes of the organelles, and they use the energy associated with ATP hydrolysis to transport the numerous substrates. A notorious member of these transporters is the p-glycoprotein, which is one of the proteins responsible for resistance development [8]. The main issue is that, with the resistance developed, patients are suffering from severe side effects with little to no cure for cancer.

Furthermore, Ding et al. demonstrated that a tocopherol called γ-tocotrienol can reverse the MDR [9]. Altun et al. demonstrated the severe side effects of chemotherapy: nausea and vomiting, fatigue, hair loss, change in taste, and constipation were the most common side effects [10]. Thus, there is a critical need to differentiate between chemotherapy-sensitive and -resistant cancer cells. Extracellular vesicles (EVs) are significant biomarkers for cancer diagnosis [11]. Evs are bilayer membrane particles that are secreted from approximately all cell types, while Evs cannot replicate as cells do [11]. There is a huge variation in EVs size from 20 nanometers to 10 μm, and the difference in EVs’ size and synthesis mechanism leads to Evs variation [11].

Exosomes are one of the most common types of EVs [11]. Exosomes are bilayer-membrane extracellular vesicles produced in most eukaryotic cells, which are the cells that contain a defined membraned nucleus. Furthermore, they are present in tissues and most biological fluids such as blood, urine, and saliva, and there is an extreme variation in the size of exosomes, as their size can be within a range from 30 to 150 nanometers [12]. Exosomes are carriers of their parent cell markers and are significantly involved in several biological functions [13]. Protein and nucleic acids are the chief components of exosomes [14]. Moreover, exosomes contain both mRNA and miRNA, which are different than intra-cellular mRNA and miRNA [15]. Exosomes are also in charge of communicating and transferring molecules from one cell to another; in doing so, they can stimulate the immune system in the body [16]. For instance, exosomal mRNA can alter the protein production in the recipient cells [17]. Likewise, exosomes can be used for diagnostic and therapeutic purposes [18,19]. Martins et al. demonstrated that exosomes can be used for Alzheimer’s disease diagnosis and as therapeutic targets [20]. Non-invasive, high-sensitivity, and rapid responses are the chief advantages of using exosomes in diagnosis [21,22]. Furthermore, exosomes are currently being considered as early diagnostic biomarkers [23]. Wang et al. illustrated that early cancer diagnosis can significantly reduce the cost of treatment of cancer; therefore, exosomes are potentially important for cancer diagnosis and treatment [24]. Therefore, there is a need for a method that is capable of determining whether cancer cells are sensitive or resistant to chemotherapy based on their exosomal content analysis. Conventional detection techniques necessitate enormous amounts of samples and extensive technical steps. As a proposed solution, exosomal RNA has been significantly used as a promising biomarker for tumor prognosis [25]. To differentiate between MCF7 and MCF7/ADR, a simple electrochemical protocol based on the MCF7 and MCF7/ADR exosomal total RNA was used, with the assumption that the different exosomal RNAs should have different composition and, hence, a different adsorption affinity towards gold. That, in turn, shall affect the gold reactivity towards a standard electrochemical reaction such as K_4_[Fe(CN)_6_]/K_3_[Fe(CN)_6_] reaction.

## 2. Results and Discussion

### 2.1. Physical Characterization of Exosomes and Isolated Exosomal RNAs

**Figure 1:**
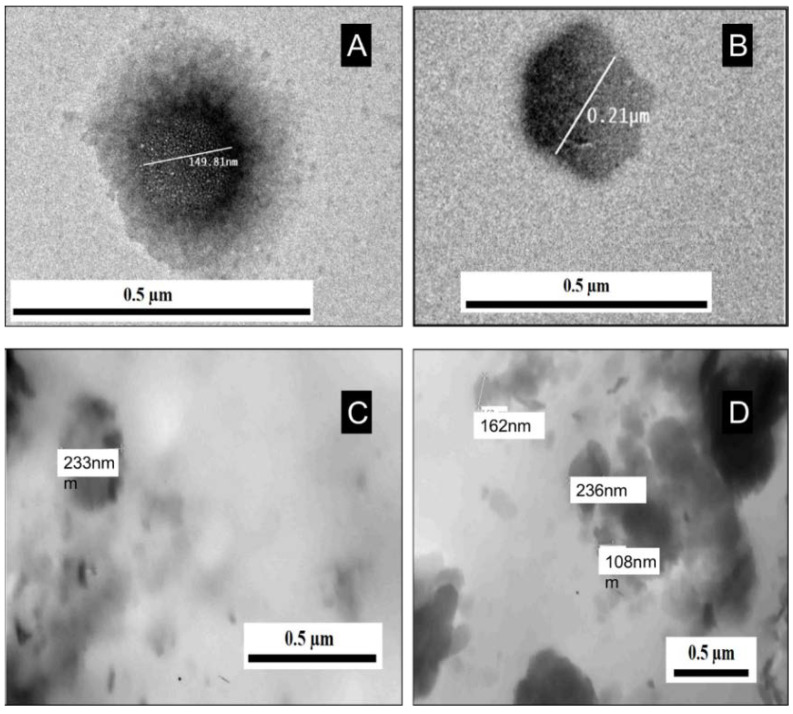
The TEM images of the exosomes isolated from (**A**) MCF 7, (**B**) MCF 7/ADR, (**C**) SH-5YSY, and (**D**) HepG2.

The TEM was used to prove the presence of the exosomes in the samples and monitor their size. Figure 1 shows the TEM images of the exosomes extracted from MCF7, MCF7/ADR, SH-SY5Y, and HEPG2 cell lines. However, the TEM images were not particularly clear, as TEM cannot be used to demonstrate the quality of exosomes, especially when the used sample is frozen [26,27]. Furthermore, the lipid bi-membrane and main exosomal components were not observed in all images. On the other hand, Figure 1 showed that the average diameter for all different exosomes was about 200 nm, confirming the presence of the exosomes in all samples. Exosomes can be found isolated or aggregated with other exosomes, as shown in Figure 1. The shape of the observed exosomes in all images is round, as clearly shown in Figure 1A. The TEM images showed almost the same morphology and size in the four types of exosomes.

**Figure 2:**
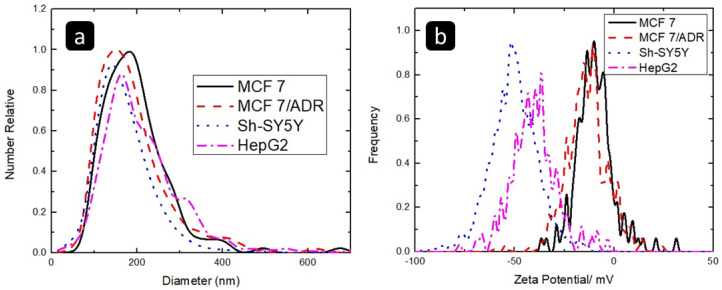
(**a**) The Zeta-sizer measurements and (**b**) The Zeta potential for the MCF7, MCF7/ADR, Sh-SY5Y, and HEPG2 isolated exosomes.

The Zeta-sizer peaks, shown in Figure 2a, for the MCF 7, MCF 7/ADR, Sh-SY5Y, and HEPG2 isolated exosomes were at 173.7 nm, 158.7 nm, 154.6 nm, and 156.9 nm, respectively, very close to diameter range of exosomes “140 to 160 nm” measured by Martins et al. [28]. The diameters of all the exosomes were close to each other, except for the exosomes isolated from MCF 7 which were slightly bigger. As shown in Figure 2a, the peak position and the size distribution of all exosomes were very close to each other. Therefore, the Zeta-sizer NTA results did not allow for differentiation of the different types of the studied exosomes [29].

The Zeta potential peaks for the MCF7, MCF7/ADR, SH-SY5Y, and HEPG2 isolated exosomes were at −11.3 mV, −11.6 mV, −51 mV, and −37.8 mV, respectively as shown in Figure 2b. The Zeta potential values of the exosomes isolated from SH-SY5Y and HEPG2 are significantly different than the ones isolated from MCF7 or MCF7/ADR and could allow for differentiation of these exosomes. However, Zeta potential cannot be used to differentiate between exosomes isolated from sensitive and resistant cell lines (MCF7 and MCF7/ADR), as they have almost the same Zeta potential value.

On the other hand, all the exosomes have a negative net charge which is similar to what Beit-Yannai et al. has mentioned [30]. The low Zeta potential value, such as the Zeta potential of MCF7 and MCF7/ADR exosomes, indicates low stability and affinity to aggregate, while the exosomes with a high net charge such as SH-SY5Y and HEPG2 tend to be more stable. The data for the Zeta-sizer analysis and Zeta potential are summarized in Table 1.

In this study, screen-printed gold electrodes will be used to electrochemically differentiate between the MCF7 and MCF7/ADR using their isolated total RNA. The surface of the gold electrode needs to be cleaned and hence allow for consistent measurements. SEM images of the gold screen-printed electrodes before and after the cleaning procedures are shown in Figure S1 (Supplementary Materials). Figure S1A–C show SEM images of the as-received gold electrode before cleaning at different magnifications. The presence of contaminants was observed, confirming the necessity for performing an electrochemical cleaning step before testing the exosomal RNA adsorption. SEM images in Figure S1D–F showed that after cleaning, the surface is smoother and cleaner, in agreement with the electrochemical results. The small white particles could be attributed to the PBS crystals that remained after washing and drying.

Since the adsorption behavior of the exosomal RNA and its effect on the gold electrode electrochemical response will be used for the electrochemical characterization of the four cell lines, the nature of the electrode before and after adding those exosomal RNAs is requested. For imaging the exosomal RNA, 0.5 µL of 25 ng/µL of each exosomal RNA solution was deposited on different cleaned gold electrodes, followed by several cycles of washing with PBS solution, to confirm that the measured electrochemical behavior is related to the adsorbed exosomal RNA. There was no need to use gold sputtering for the FE-SEM imaging after depositing the RNA at the surface of the gold electrode.

**Figure 3:**
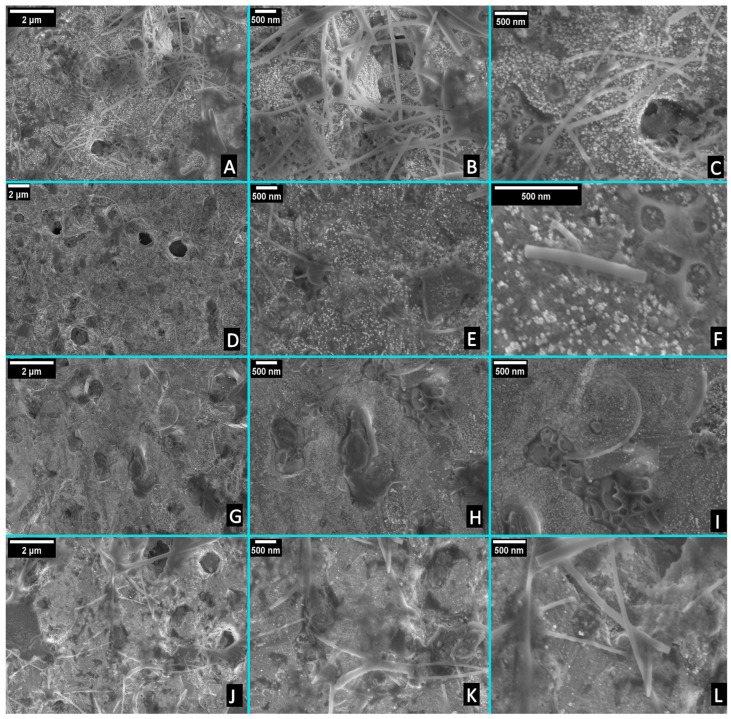
The SEM images for the exosomal RNAs that were isolated from; (**A**–**C**) MCF7, (**D**–**F**) MCF7/ADR, (**G**–**I**) SH-SY5Y, and (**J**–**L**) HEPG2.

Figure 3 shows the SEM images for the four different types of exosomal RNAs. SEM is commonly used to reveal the morphology, as well as the size distribution of the exosomal RNAs. As shown in Figure 3, the tested exosomal RNAs adsorbed on the gold surface have the shape of a rod. The average thickness of the exosomal RNA isolated from MCF7 was 55 nm, while it was 45 nm for the exosomal RNA isolated from MCF7/ADR. Furthermore, the average thickness of the exosomal RNA isolated from SH-SY5Y was 50 nm, and the average thickness of the exosomal RNA isolated from HEPG2 was 69 nm, which is significantly larger than other exosomal RNA. The size (diameter) distribution of the exosomal RNA is shown in Figure S2.

Further magnification has been applied to clearly show the exosomal RNA. The concentration was 25 ng/uL for all exosomal RNAs, while the adsorbed percentage of the RNA on the gold surface was different. The quantity and distribution of the exosomal RNA were not identical in the four samples. For instance, a high percentage of the exosomal RNA isolated from MCF7 was adsorbed on the gold surface relative to the exosomal RNA isolated from MCF7/ADR. Moreover, the quantity of the exosomal RNA isolated from SH-SY5Y was too low, as shown in Figure 3G–I. On the other hand, SEM images have the same structure for the different exosomal RNAs; therefore, SEM cannot be used to differentiate between the different exosomal RNAs.

FTIR characterization, shown in Figure S3, aimed to confirm that the total RNA extraction from the designated exosomes was successful and the samples were not contaminated. According to Figure S3, intense broadband at around 3300 cm^−1^ was observed for all the exosomal RNAs, which could be assigned to the overlap of the NH and OH bands, which theoretically fall at 3300–3500 cm^−1^ and 3200–3550 cm^−1^, respectively [31,32,33]. Another band located at 1640 cm^−1^ was observed and could be attributed to the carbonyl group, which theoretically falls between 1600–1900 cm^−1^ [32]. Moreover, there was a band at 2127 cm^−1^ which could be attributed to alkyne, as it is weak in the range 2100–2250 cm^−1^ [34]. The FTIR results for the four exosomal RNAs in Figure S3 showed almost identical peaks.

### 2.2. Electrochemical Characterization

According to the previous results, the Zeta potential of the isolated exosomes could allow for differentiation of the SH-SY5Y, HEPG2, and MCF7 or MCF7/ADR, but was not sufficient to differentiate between the sensitive and resistant cell lines (MCF7 and MCF7/ADR). The FT-IR results confirm that the total RNA extraction from the designated exosomes was successful, and the samples were not contaminated. In addition, the SEM images confirm the adhesion of the RNAs to the gold surface even after washing, indicating the strong adsorption of the RNAa at the gold surface and the stability of the electrode during the subsequent electrochemical tests. Herein, we tried to use the adsorption properties of the exosomal RNAs on the gold surface to differentiate between the different four cell lines under study by monitoring any changes to the ferrocyanide/ferricyanide standard redox reaction at the surface screen-printed gold electrodes after allowing the exosomal RNA to adsorb on the surface. Four different electrochemical techniques, Cyclic Voltammetry (CV), Square Wave Voltammetry (SWV), Normal Pulse Voltammetry (NPV), and Differential Pulse Voltammetry (DPV), were used. These four different types of electrochemical techniques were investigated to find the technique that would give the best differentiation between the total RNA behaviors and the lowest LOD value. In our essay, the electrode was subjected to surface modification with the adsorbed total RNA at the gold surface. The electrochemical responses of SH-SY5Y and HEPG2 exosomal RNAs are shown in Figures S4 and S5, respectively. As shown in Figure S4, there was current suppression in all four techniques relative to the blank due to the adsorption of the SH-SY5Y exosomal RNA, but there was no linearity with the concentration range.

Similarly, Figure S5 shows no trend with the HEPG2 exosomal RNA increase in concentration, with no significant peak current nor peak potential change. We tested different cells’ exosomal RNA. The neuroblastoma Sh-Sy5y and the hepatoblastoma HepG2 cells and MCF7 cells were all examined. The responses obtained could not discriminate between these cells based on their exosomal RNAs (Figures S4 and S5). If this was simply an interaction between a bare electrode and a solution of biomolecules, we should have seen differences between these three cells in addition to MCF7/ADR; however, this was not the case. Given that we could not discriminate between the different cells tested using different electrochemical characterizations, detecting the electrochemical behavior of the HEPG2 and SH-SY5Y exosomal RNA in the subsequent techniques was not investigated further. Conversely, both MCF7 and MCF7/ADR exosomal RNAs showed significant changes; hence, they were studied in further detail, as seen below.

#### 2.2.1. Electrochemical Characterization of Exosomal RNA Isolated from MCF7

**Figure 4:**
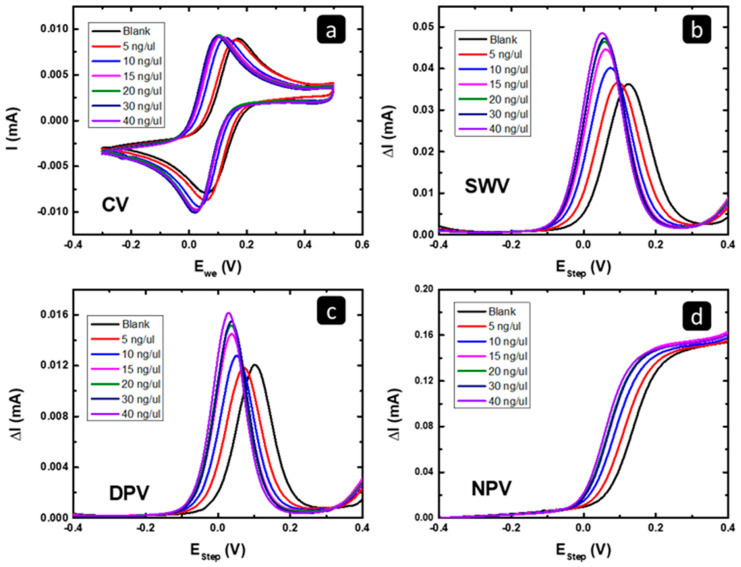
The electrochemical responses of Au-SPE before and after depositing different amounts of the MCF7 exosomal RNA using (**a**) CV, (**b**) SWV, (**c**) DPV, and (**d**) NPV techniques.

The electrochemical responses in Figure 4A–D show the effect of increasing the amount of the deposited MCF7 exosomal RNA on the gold response towards Fe(CN)_6_^4+^/Fe(CN)^3+^ reaction using cyclic voltammetry (CV), square wave voltammetry (SWV), differential pulse voltammetry (DPV), and normal pulse voltammetry (NPV). Relative to the blank response, a shift in the peak potential (or the limiting current onset potential) to lower values and a slight increase in the peak/limiting current in all four techniques were observed. That behavior should be related to the change in the surface nature of the electrode material due to the adsorption of the MCF7 exosomal RNA, which changed the surface of the gold electrode and its activity towards the Fe(CN)_6_^4+^/Fe(CN)^3+^ reaction, causing an enhancement in the electron transfer due to the higher conductivity of the MCF7 exosomal RNA adsorbed on the electrode surface [35].

**Figure 5:**
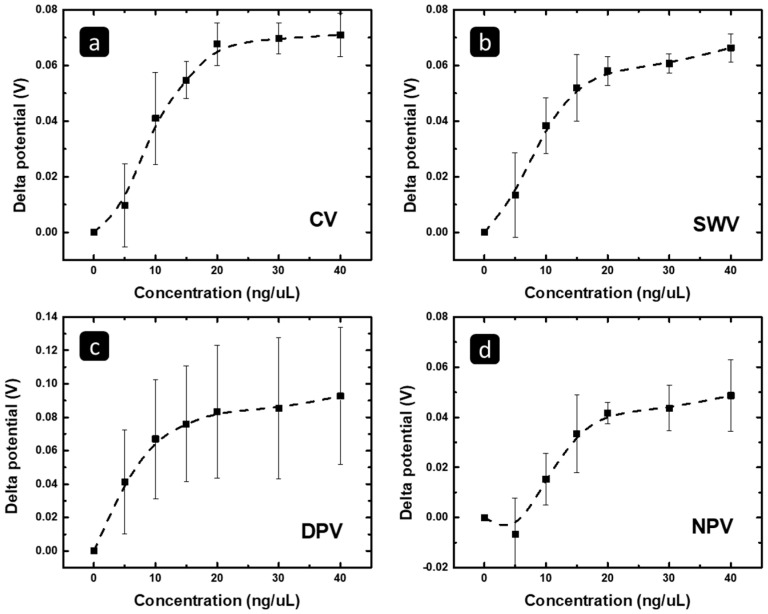
The correlation between the (**a**) CV, (**b**) SWV, (**c**) DPV, and (**d**) NPV potential change versus the concentration (deposited amount) of the MCF 7 exosomal RNA.

The peak potential change was tracked versus the concentration of the exosomal MCF 7 RNA for the four techniques and presented in Figure 5, using a concentration range of 0–40 ng/uL. Figure 5 shows that the peak potential shifts to less positive values significantly and linearly with the exosomal RNA concentration up to 20 ng/μL, and then reaches a plateau, indicating the insignificant change in peak potential with increasing concentration. This behavior should be related to the surface saturation with exosomal RNAs, and hence, a further increase in the exosomal RNAs concentration will not lead to further adsorption of these exosomal RNAs.

In addition, it could be seen in Figure 5 that DPV has high error bar values in comparison to the other techniques. Additionally, in the case of CV and SWV, we could observe that the error bar values reduce as the concentration of the exosomal RNA increases, especially when the plateau is reached.

**Figure 6:**
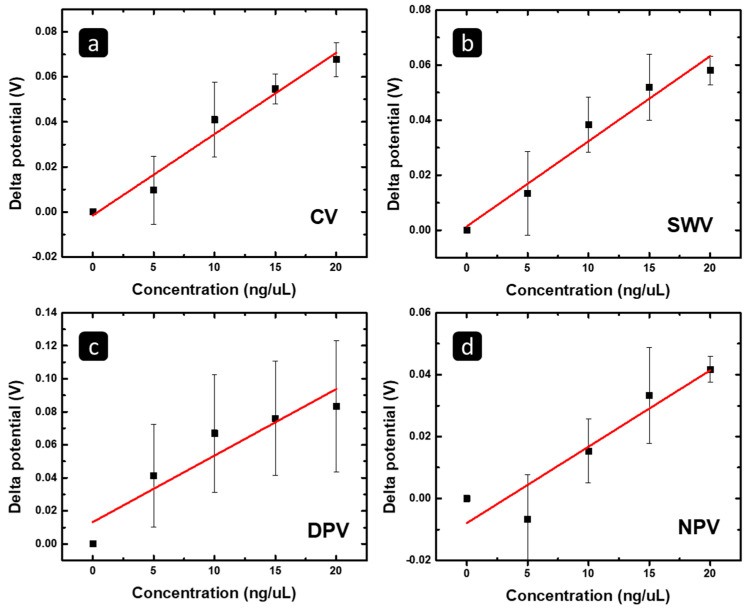
The correlation between the (**a**) CV, (**b**) SWV, (**c**) DPV, and (**d**) peak potential change versus the concentration of the MCF7 exosomal RNA, using the concentration range of 0–20 ng/μL, in which a linear behavior is observed.

On the other hand, to highlight the concentration range in which the response has linearity with the MCF 7 RNA concentration, Figure 6 shows the correlation between (a) the CV peak potential shift, (b) the SWV peak potential shift, (c) the DPV peak potential shift, (d) the NPV peak potential shift, and the concentration of the isolated exosomal RNA from MCF7 in the concentration range of 0–20 ng/μL. The R^2^ and a limit of detection (LOD) based on the linear fit of the data are summarized in Table 2.

Furthermore, to further investigate the validity of the results, statistical analysis was performed. The test of one-way ANOVA showed that the results for the CV and SWV were significant with a *p*-value less than 0.001, which means that the results were not reached by chance. Furthermore, the post hoc (Tukey) test showed that the experiment was significant at all concentrations, and the post hoc test showed that the results were significant at all detected concentrations, except at 5 ng/µL for the CV and SWV. On the other hand, the tests of one-way ANOVA and the post hoc test for the DPV showed that the results were not significant. Furthermore, for the NPV, the ANOVA test showed significant results, too; the *p*-value was less than 0.001, and the post hoc test showed that the results were significant at concentrations of 15 and 20 ng/µL. According to these results and a statistical analysis, the change in the peak potential of the CV, SWV, and NPV responses is suitable to follow/monitor the concentration (amount) of MCF7 exosomal RNA to 20 ng/µL with high linearity and sensitivity, showing significant results, while the DPV response is not suitable.

**Figure 7:**
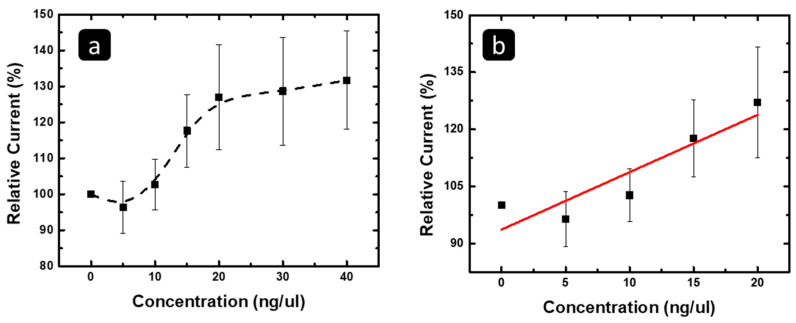
The correlation between the relative increase of the SWV peak current versus the concentration of the MCF 7 exosomal RNA with the concentration range of (**a**) 0–40 and (**b**) 0–20 ng/µL.

Furthermore, there was a slight increase in the peaks of most of the techniques, while after data analysis, we observed that there was no linear correlation between the increase in the peaks with the concentration change in most of the techniques. Notably, SWV showed a trend for the peak current increase with increasing concentration, as shown in Figure 7a, with linearity in the concentration range of 0–20 ng/µL before a plateau was reached. Focusing on the linear range, Figure 7b shows the linear fit of the data, with the R^2^ and the limit of detection being 0.78 and 41.5 ng/µL (Table 2). The ANOVA test showed that the experiment was significant, as the *p*-value was less than 0.01, while the post hoc Tukey test showed that all concentrations were nonsignificant, except for the concentration of 20 ng/µL. Therefore, SWV at a concentration of 20 ng/µL can be used to detect the exosomal RNA isolated from MCF 7, while the limit of detection is relatively higher than those of the potential differences.

#### 2.2.2. Electrochemical Characterization of Exosomal RNA Isolated from MCF7/ADR

**Figure 8:**
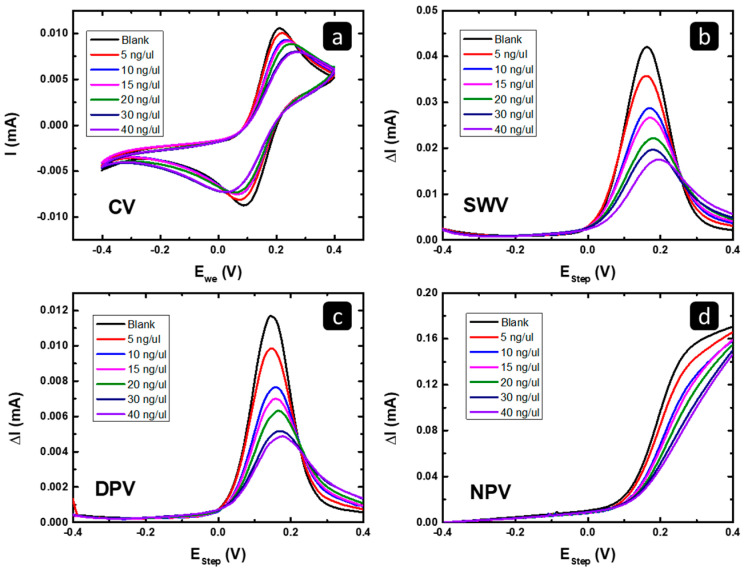
The electrochemical responses of the gold SPE before and after depositing different amounts of the isolated exosomal RNA from MCF7/ADR using (**a**) CV, (**b**) SWV, (**c**) DPV, and (**d**) NPV.

The electrochemical responses in Figure 8 shows the effect of increasing the amount of the deposited MCF7/ADR exosomal RNA on the gold response towards Fe(CN)_6_^4+^/Fe(CN)^3+^ reaction using cyclic voltammetry (CV), square wave voltammetry (SWV), differential pulse voltammetry (DPV), and normal pulse voltammetry (NPV). As shown in Figure 8, there is current suppression in all four techniques relative to the blank curve “the black curve” and peak potential shift towards more positive values (slower kinetics). This behavior has been related to the inhibition of reactant interaction (e.g., π−π interactions, hydrogen bonds, and/or electrostatic interactions) with the electrode surface [36]. In this study, the suppression should be the result of the adsorption of the MCF7/ADR exosomal RNA on the gold working electrode, which, in contrast with MCF7, decreased the active surface area of the working electrode and changed it to be less active towards the Fe(CN)_6_^4+^/Fe(CN)^3+^ reaction.

According to Equations E1–E4 (SI), the current/current difference, in the four techniques, is surface area (A) dependent and assumes fast kinetics, as all the remaining conditions were constant. Therefore, this suppression should be related to lowering the electrochemical activity of the electrode or decreasing the electrochemical active site of the working electrode because of the adsorption of the MCF7/ADR exosomal RNA [37,38].

**Figure 9:**
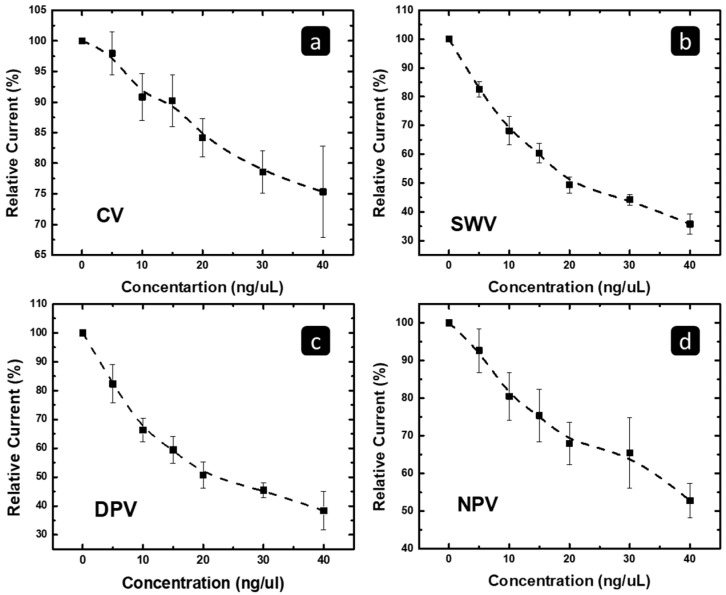
The correlation between relative suppression of the peak current of the (**a**) CV, (**b**) SWV, (**c**) DPV, and (**d**) NPV techniques versus the concentration of the MCF7/ADR exosomal RNA.

The peak current change was tracked versus the concentration of the exosomal MCF 7/ADR RNA for the four techniques and presented in Figure 9, using a concentration range of 0–40 ng/uL. Figure 9 shows that the peak current decreases significantly and linearly with the exosomal RNA concentration up to 20 ng/uL, and then becomes less significant. This behavior should be related to the increase in lateral interaction between the adsorbed RNA, and hence, reduced probability of extra coverage when the exosomal RNA concentration is higher than 20 ng/uL. In addition, it can be seen in Figure 9 that the NPV technique has high error bar values in comparison to the other techniques, with the SWV technique showing the smallest error bar values.

**Figure 10:**
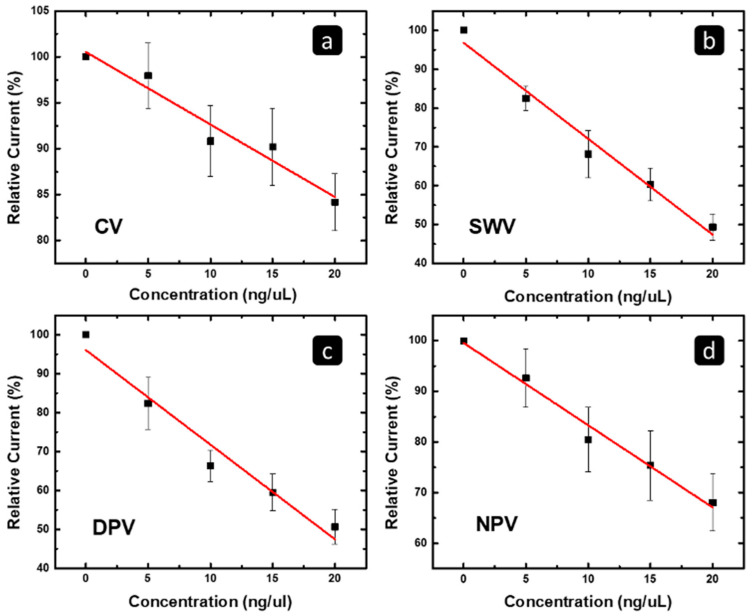
The correlation between the relative suppression of the peak current of the (**a**) CV, (**b**) SWV, (**c**) DPV, and (**d**) NPV techniques versus the concentration of the MCF7/ADR exosomal RNA, using a concentration range of 0–20 ng/µL in which a linear behavior is observed.

On the other hand, to highlight the concentration range in which the response has linearity with the concentration of the MCF7/ADR RNA, Figure 10 shows the correlation between (a) the CV, (b) SWV, (c) DPV, (d) NPV peak current/limiting current and the concentration of the MCF7/ADR RNA, at the concentration range of 0–20 ng/uL. The R^2^ and a limit of detection (LOD) based on the linear fit of the data are summarized in Table 3. The SWV gave the best R^2^ of 0.97; the lowest limit of detection was “4.8 ng/µL”, and the most significant concentration was 20 ng/µL for the MCF7/ADR exosomal RNA Detection. All the MCF7/ADR results, regardless of the technique, were nonsignificant for the concentration range from 20 to 40 ng/µL due to the high value of the error bar at this range of concentration. The error bars showed a high degree of overlap, which reflects the uncertainty in the correlation between the concentration and responses.

Furthermore, to further investigate the validity of the results, statistical analysis was performed. The test of one-way ANOVA showed for the CV, SWV, DPV, and NPV that the results were significant with a *p*-value less than 0.001, which means that the results were not reached by chance. Furthermore, the post hoc (Tukey) test showed that the experiment was significant at all detected concentrations, except at 15 ng/µL for the CV and 5 ng/µL for the NPV.

According to these results and the statistical analysis, the relative suppression of the peak current of the CV, SWV, DPV, and NPV responses is suitable to follow/monitor the concentration (amount) of MCF7/ADR exosomal RNA to 20 ng/µL with high linearity and sensitivity, showing significant results.

According to the results of the MCF7 and MCF7/ADR exosomal RNAs, the key difference between them is that the exosomal MCF7 increased the peak current of the standard K_4_[Fe(CN)_6_]/K_3_[Fe(CN)_6_] electrochemical reaction and shifted the peak potential to less positive values. In contrast, the exosomal MCF 7/ADR decreased the peak current with a slight peak potential shift to more positive values. These differences could be used to differentiate between the two exosomal RNAs and, hence, their original cells. This specificity is useful and simple to distinguish between the MCF and MCF7/ADR cells and could be further extended to distinguish between breast cancer sensitivity to chemotherapy in the future.

## 3. Materials and Methods

### 3.1. Materials

The breast adenocarcinoma MCF7 cell line (ATCC^®^HTB-22™), Doxorubicin-resistant breast cancer MCF7/ADR, hepatocellular HEPG2 [HEPG2] cell line (ATCC^®^HB-8065™), and neuroblastoma SH-SY5Y cell line (ATCC^®^CRL-2266™) were obtained from Nawah (Cairo, Egypt). Dulbecco’s Modified Eagle Medium (DMEM) High Glucose (41965-039), Roswell Park Memorial Institute (RPMI) 1640 and fetal bovine serum (FBS) (10270-106) were obtained from Thermo Fisher Scientific (Waltham, MA, USA). Dimethyl sulfoxide (DMSO) (67-68-5), penicillin–streptomycin mixture (09-757F), and phosphate-buffered saline (10×) PBS (17-516Q) were obtained from Lonza-Bioscience (Basel, Switzerland). RNeasy Kits for RNA isolation were obtained from Qiagen (Hilden, Germany). Gold electrode (screen printed electrode (BVT-AC1) electrochemical sensors with silver and silver chloride mixed as reference electrodes were also purchased (Utrecht, The Netherlands).

### 3.2. Cell Culture

MCF7, MCF7/ADR, SH-SY5Y, HEPG2 cells were grown in 75 cm^2^ flasks until 70% confluence at 37 °C in a 5% CO_2_ humidified incubator. For exosomes isolation and preparation, MCF7, MCF7/ADR, and HepG2 cells were cultured in DMEM supplemented with 1% penicillin–streptomycin and 10% Fetal Bovine Serum (FBS). Cells were passaged three times before exosome isolation. On reaching 70% confluency, cells were harvested by trypsinization and replated in serum-free DMEM. Similarly, SH-SY5Y cells were cultured in RPMI supplemented with 1% penicillin–streptomycin and 10% FBS. On reaching 70% confluency, cells were harvested by washing and replated in serum-free RPMI. Following a 48 h incubation period, the culture media were collected and centrifuged for isolation of exosomes. Cells were counted following media collection.

### 3.3. Exosomes Isolation by Ultracentrifugation and Physical Characterization

The isolated culture media were transferred to 50 mL centrifuge tubes and spun at 2000× *g* for 20 min at 4 °C. Next, the supernatants were transferred to fresh tubes and ultracentrifuged at 100,000× *g* for 70 min at 4 °C. In cases in which the sample volume was less than 75% of the tube volume, PBS was added to compensate for the remaining volume. Pellets containing exosomes were resuspended in 1 mL PBS, each, and the resuspended pellets were pooled together and filled to volume with PBS. The pooled samples were then ultracentrifuged at 100,000× *g* for 1 h at 4 °C. The supernatant was completely removed, and the pellets were resuspended in 50–100 μL of PBS. Exosome samples were stored in 100 μL aliquots at −80 °C until further processing.

For TEM imaging, the following procedure was used; in 1 ml 2.5% glutaraldehyde at pH 7, the exosomal pellet was dissolved and redissolved in 0.1 M sodium cacodylate solution for one hour. Following removal of the fixative, the pellet was washed three times in 1 mL 0.1 M sodium cacodylate buffer at 25 °C for 10 min, each, followed by incubation in 1 mL of 2% osmium tetroxide for 60 min at 4 °C. Once again, the fixative was removed and the pellet was washed in 0.1 M sodium cacodylate buffer three times, 10 min each. The samples were subsequently dried with graded acetone series. Acetone was added to low-viscosity embedding medium series (3:1, 1:1, 1:3). Afterward, samples were incubated with low-viscosity embedding medium (100%). The samples were embedded using an embedding mold at 4 °C and baked for one day. The embedded samples were then cut into 60 nm thick sections using an ultra-microtome. Thereafter, the sections were stained with 2% uranyl acetate and lead citrate for 20 and 10 min, respectively. Finally, the samples were observed under the grid using JEOL^®^ (Peabody, MA, USA) TEM model JEM-1200EX at 80 kV.

For the Zeta-size and Zeta-potential measurements, after the successful isolation of the exosomes, exosomes were diluted 1 to 200 by PBS. The Zeta-size and Zeta-potential for the four different exosomes: MCF 7 exosomes, MCF 7/ADR exosomes, Sh-sy5y exosomes, and HepG2 exosomes were measured and analyzed using ZetaView S/N 21-619, Software ZetaView (version 8.05.14 SP7) (Particle Metrix) [37].

### 3.4. RNA Isolation from Exosomes

One mL of Qiazol Lysis Reagent was added to 200 μL of each sample and mixed by vortexing. The homogenate was allowed to stand at room temperature for 5 min to stimulate the segregation of the nucleoprotein complexes, then 200 μL chloroform was added and vortexed in. Following a brief incubation, the mixture was centrifuged at 12,000× *g* for 15 min, at 4 °C. The upper aqueous RNA-containing layer was then transferred to a clean tube. Next, 1.5 volumes of 99% ethanol was added to the aqueous phase and 700 μL were transferred to the Qiagen RNeasy column and centrifuged at 8000× *g* for 45 s. After discarding the flow-through, the column was reused to process the remainder of each respective sample until the entire sample has been processed. Afterward, 700 μL of RWT buffer was added to the column and centrifuged at 8000× *g* for 45 s. Further, 500 μL of RWE buffer was then added to the column and centrifuged at 8000× *g* for 45 s. Subsequently, 500 μL of 80% ethanol was added and centrifuged at 8000× *g* for 2 min. The column was then transferred to a new collection tube and centrifuged again at 15,000× *g* for 2 min. Following an additional transfer of the column to a new collection tube, 700 μL of RNase-free water was pipetted onto the column and centrifuged at 14,000× *g* for 1 min. The RNA elute was stored at −80° C until further analysis.

### 3.5. Setup and Conditions for the Electrochemical Detection of Exosomal RNA

Boriachek et al. used DPV to measure the [Fe(CN)_6_]^4−/3−^ redox system by using gold SPE, carrying out direct loading of a specific type of the miRNA into gold SPE, and comparing the results before the adsorption of miRNA and after the loading of miRNA [36]. We have modified this system by using exosomal RNA to electrochemically detect any differences between the four exosomal RNAs understudied by four different electrochemical techniques: CV, SWV, DPV, and NPV. In these techniques, we used an electrochemical active redox reaction of K_4_[Fe(CN)_6_]/K_3_[Fe(CN)_6_], using 2.5 mM of K_4_[Fe(CN)_6_] + 1× PBS buffer solution (pH = 7.4) at a screen-printed electrode (SPE, BVT-AC1.W1.R2 Dw = 2). The SPE consists of WE (working electrode) and CE (counter electrode) of gold and the RE (reference electrode) of chlorinated silver. To test the effect of the four exosomal RNAs on the gold electrode electrochemical response towards the Fe(CN)_6_^4+^/Fe(CN)^3+^ reaction, 0.5 µL of exosomal RNA solution, with a concentration of 5 ng/µL, was deposited on the WE to add 2.5 ng of the exosomal RNA to the gold surface in each deposition step. The exosomal RNA solution was left to dry at room temperature and then its electrochemical response was measured in 2.5 mM of K_4_[Fe(CN)_6_] + 1× PBS buffer solution (pH = 7.4) using the same electrochemical techniques. To test the effect of the exosomal RNA concentration, a sequential deposition of 2.5 ng of the exosomal RNA was used on the same electrode with no cleaning in between, causing a build-up of the exosomal RNA on the WE surface. Finally, the data were analyzed to study the different exosomal RNAs behaviors and compare them to the standard behavior of the WE electrode in the absence of any exosomal RNA.

Initially, we observed that the SPE gold electrode did not present the expected K_4_[Fe(CN)_6_]/K_3_[Fe(CN)_6_] standard electrochemical response. Therefore, we had to develop a cleaning protocol in which 50 CVs with a 100 mV/s scan rate and a potential range of −1.2 V to 0.6 V in 1× PBS buffer (pH = 7.4) were performed. Afterward, the electrochemical response of the electrode was tested in 2.5 mM of K_4_[Fe(CN)_6_] + 1× PBS buffer solution (pH = 7.4) by applying several CVs with a 20 mV scan rate, and the potential range from −0.3 V to 0.5 V until the accepted peaks were reached. For further testing, only the cleaned gold SPE with acceptable behavior was used.

**Figure 11:**
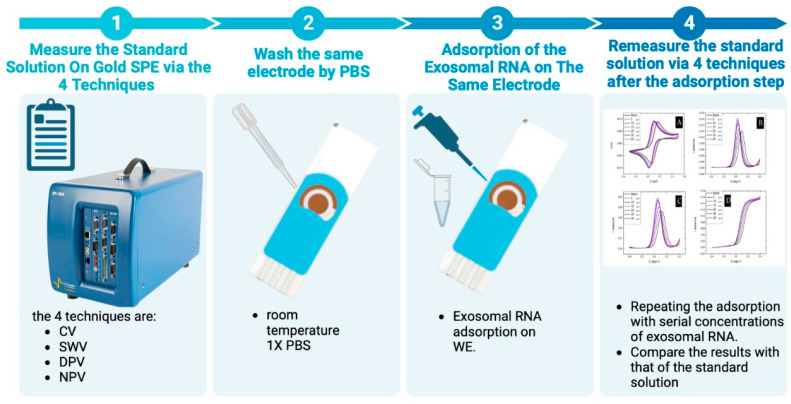
A diagram showing the procedure for measuring the effect of exosomal RNA adsorption on the Au electrode surface, using the standard K_4_[Fe(CN)_6_]/K_3_[Fe(CN)_6_] electrochemical reaction.

For all the electrochemical measurements, IR compensation was applied. The results are triplicates; the limit of detection (LOD) was calculated according to Equation E5 (SI), and error bars were used in each concentration of each graph. All the techniques were measured sequentially on the same electrode. Figure 11-1 measuring the standard K_4_[Fe(CN)_6_]/K_3_[Fe(CN)_6_] electrochemical solution by four different technique; the first technique in the experiment was CV using three cycles with a 20 mV/s scan rate and a potential range of −0.3 to 0.5 V, the second technique was SWV with a potential range of −0.4 to 0.4 V, step height of 10 mV, pulse height of 50 mV, and pulse width of 100 ms, the third technique was NPV with a potential range of −0.4 to 0.4 V, a pulse height of 5 mV, a pulse width of 80 ms, and a pulse time of 100 ms, and the final technique was DPV with a potential range of −0.4 to 0.4 V, a step height of 10 mV, a pulse height of 25 mV, and a pulse width of 100 ms. Figure 11-2 shows washing the electrode by 1xPBS after the four techniques measurements. Then, in Figure 11-3 adsorbing the exosomal RNA on the surface of the working electrode.

Finally, Figure 11-4 shows The effect of the exosomal RNA adsorption on the electrochemical behavior of the Au electrode towards the K_4_[Fe(CN)_6_]/K_3_[Fe(CN)_6_] electrochemical reaction was plotted versus the RNA concentration. For instance, the MCF7/ADR exosomal RNA resulted in a peak current suppression; therefore, we have plotted the percentage of the current at the concentration to the standard solution (relative current) versus the concentration. Then, we calculated the standard deviation of the number of trials that were triplicated to be considered as the error bars of our graphs. This was followed by statistical analysis to determine the best technique with the highest linearity and lowest sensitivity using R^2^, the limit of detection, ANOVA, and Tukey tests. R^2^ indicates the linearity, as the closest number to one means the highest linearity, while the limit of detection is used to test the sensitivity of the technique. R^2^ and limit of detection and all graphs were developed using Origin software, while Anova and the post hoc (Tukey) tests were developed using SigmaPlot software. Furthermore, Anova and the post hoc (Tukey) tests measured the significance of the experiment and the significance at each concentration, respectively. If the *p*-value in the Anova test was less than 0.005, the experiment was significant and the result was not reached by chance, whereas the post hoc (Tukey test) directly states if the experiment at this concentration is significant or not.

## 4. Conclusions

Regarding the exosome’s characterization, TEM showed the exosomes isolated from the four different cell lines, while TEM images cannot differentiate between the different types of exosomes. Furthermore, Zeta-sizer cannot be used to differentiate between the different exosomes, as they have very close average diameters. Zeta potential can be used to differentiate between MCF7, Sh-SY5Y, and HepG2. However, Zeta potential cannot be used to differentiate between MCF 7 and MCF 7/ADR. On the other hand, the exosomal RNA characterization using FTIR showed identical peaks for the exosomal RNAs isolated from the four different cell lines. Therefore, FTIR cannot differentiate between the different types of exosomal RNAs. Moreover, SEM showed the same structure of the exosomal RNA in the four samples; therefore, SEM images cannot differentiate between the different types of exosomal RNA. Finally, the adsorption behavior of the studied cells’ exosomal RNAs on the Au electrode successfully allowed for electrochemical differentiation between the exosomal RNAs of MCF 7 and MCF7/ADR, while it could not differentiate exosomal RNAs of Sh-SY5Y and HepG2.

The system compares the gold screen-printed electrodes using four electrochemical techniques on the active electrochemical standard solution potassium ferrocyanide and potassium ferricyanide with the same solution after adsorption of the exosomal RNA on the exosomal RNA isolated from MCF 7 showed a significant peak current shifting to the lower potential and a slight increase in the peak current relative to potassium ferrocyanide and potassium ferricyanide solution. However, the exosomal RNA isolated from MCF 7/ADR showed significant peak current suppression relative to potassium ferrocyanide and potassium ferricyanide solution. The concentration ranges from 0 to 20 ng/µL showed the best statistical and linearity results for exosomal RNA isolated from MCF 7 and MCF 7/ADR; therefore, this range will be optimized for further research.

## Figures and Tables

**Table 1:** The summary of the Zeta-sizer and Zeta potential peaks for the four exosomes isolated from MCF7, MCF7/ADR, SH-SY5Y, and HEPG2.

Cell	Exosomes Size Based on Zetasizer	Zeta Potential
MCF7	173.7 nm	−11.3 mV
MCF7/ADR resistant	158.7 nm	−11.6 mV
SH-SY5Y	154.6 nm	−51.0 mV
HEPG2	174.1 nm	−73.8 mV

**Table 2:** The summary of LODs based on the employed electrochemical techniques 0–20 ng/µL range of the MCF 7 exosomal RNAs.

Parameter	Electrochemical Technique				
	CV	SWV	DPV	NPV	SWV (Peak Current)
R^2^	0.96	0.95	0.84	0.84	0.78
LOD	9.1	7.4	42.2	7.2	41.5

**Table 3:** The summary of LODs based on the employed electrochemical techniques for ranges 0–20 ng/µL of the MCF 7/ADR exosomal RNAs.

Parameter	Electrochemical Technique			
	CV	SWV	DPV	NPV
R^2^	0.93	0.97	0.95	0.98
LOD	16.9	4.8	7.9	14.9

## Data Availability

The raw data supporting the conclusions of this article will be made available by contacting the corresponding authors without unjustified reservation.

## Supplementary Materials

The following supporting information can be downloaded at: https://www.mdpi.com/article/10.3390/ph16040540/s1, Figure S1: The SEM images of gold screen-printed electrodes; Figure S2: The size (diameter) distribution for the exosomal RNA; Figure S3: The FTIR spectra for the exosomal RNAs; Figure S4: The electrochemical responses of Au-SPE before and after depositing different amounts of the Sh-SY5Y exosomal RNA; Figure S5: The electrochemical responses of Au-SPE before and after depositing different amounts of the HepG2 exosomal RNA.
