# Supplementary material for: A Novel Electrochemical Differentiation between Exosomal-RNA of Breast Cancer MCF7 and MCF7/ADR-Resistant Cells
Source: Pharmaceuticals (Basel). 2023 Apr 4;16(4):540. doi: 10.3390/ph16040540 (PMC10145523; doi:10.3390/ph16040540)
Supplement: Supplementary file 1 [file pharmaceuticals-16-00540-s001.zip › pharmaceuticals-2178906-supplementary.pdf]

Supporting Information

A Novel Electrochemical Differentiation Between Exosomal-RNA of Breast Cancer MCF7 and MCF7/ADR-Resistant Cells

Mohammed H. Abdelaziz[1,2], Ehab N. El Sawy[1*], and Anwar Abdelnaser[2*]

1. Chemistry Department, School of Sciences and Engineering, The American University in Cairo, New Cairo, Egypt

2. Institute of Global Health and Human Ecology, The American University in Cairo, New Cairo, Egypt

* Correspondence: ehab.elsawy@aucegypt.edu; anwar.abdelnaser@aucegypt.edu

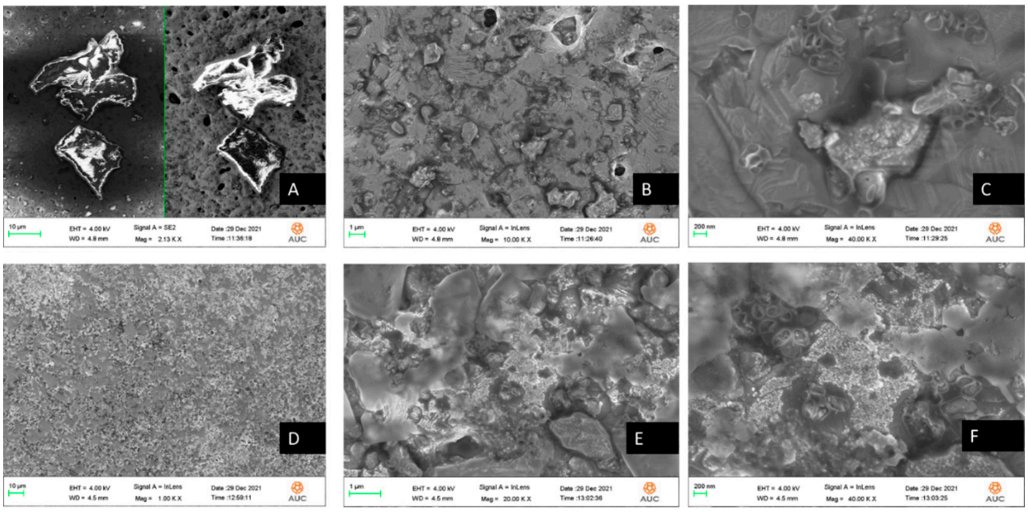

Figure S1. The SEM images of gold screen-printed electrodes; (A), (B), and (C) before cleaning, and (D), (E), and (F) after the cleaning procedures.

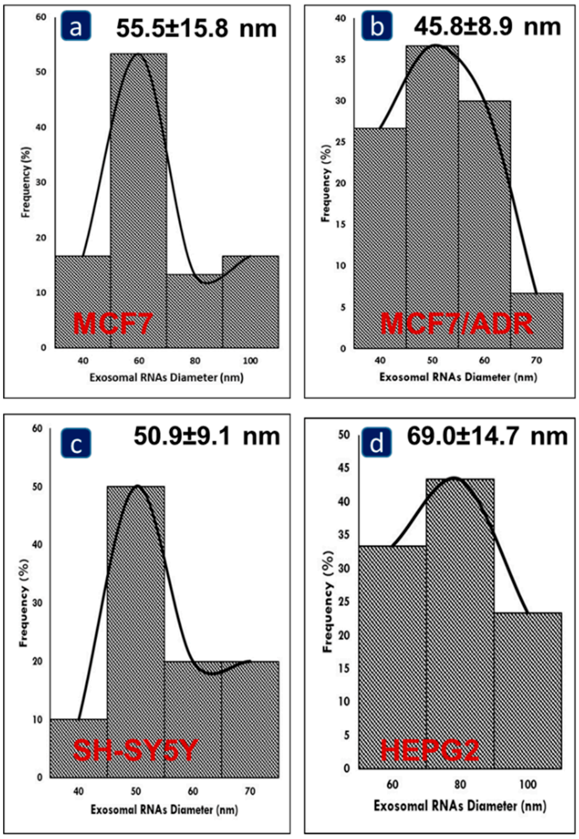

Figure S2. The size (diameter) distribution for the exosomal RNA isolated from (a) MCF7, (b) MCF7/ADR, (c) Sh-SY5Y, and (d) HEPG2.

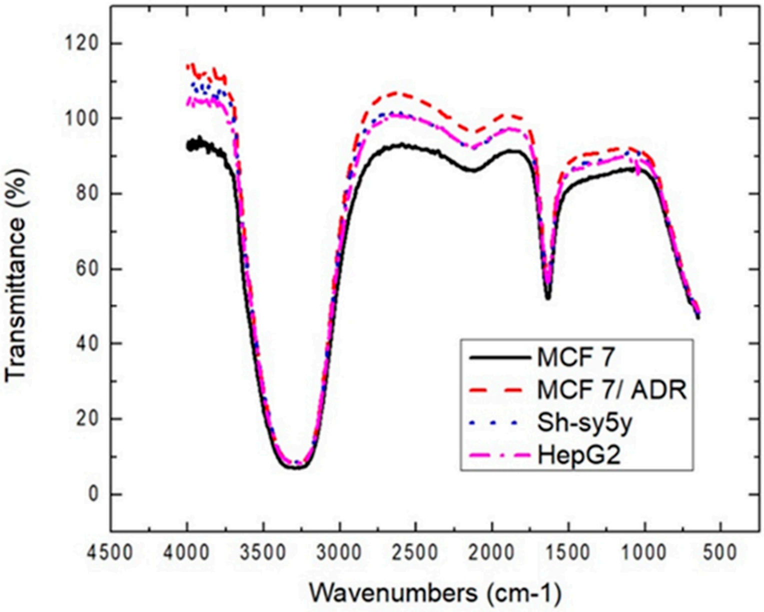

Figure S3: The FTIR spectra for the exosomal RNAs isolated from MCF7, MCF7/ADR, Sh-SY5Y, and HEPG2.

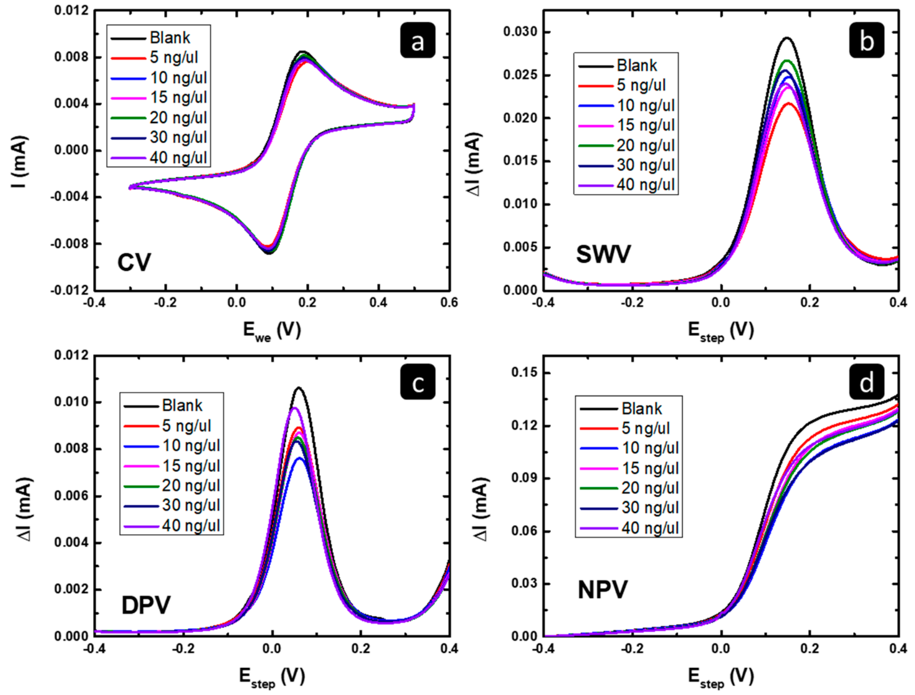

Figure S4. The electrochemical responses of Au-SPE before and after depositing different amounts of the Sh-SY5Y exosomal RNA using (a) CV, (b) SWV, (c) DPV, and (d) NPV techniques

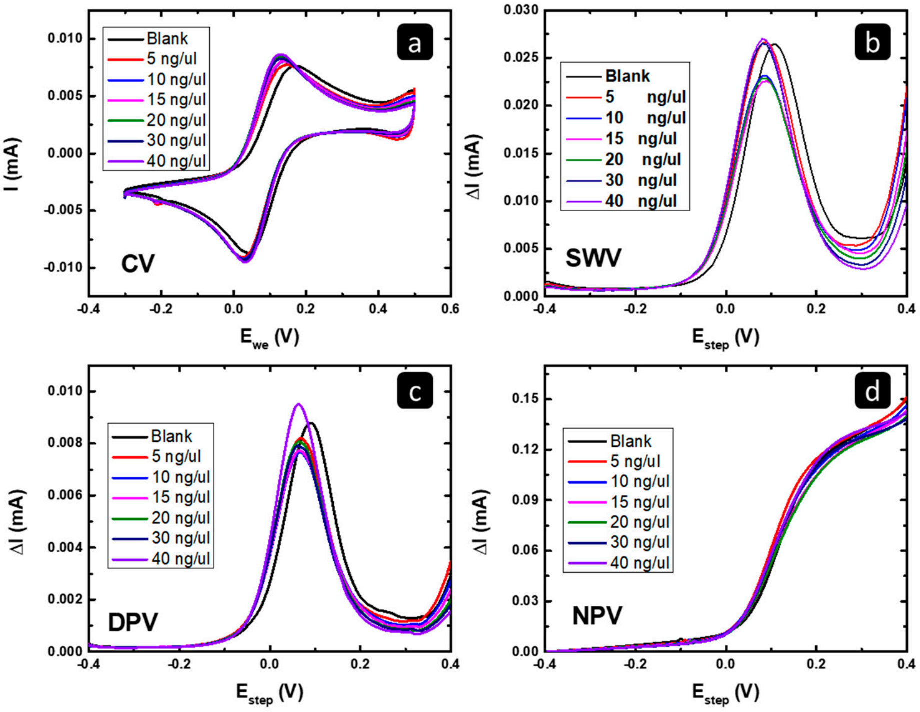

Figure S5. The electrochemical responses of Au-SPE before and after depositing different amounts of the HepG2 exosomal RNA using (a) CV, (b) SWV, (c) DPV, and (d) NPV techniques.

Equations

$$i_p = 2.69 \times 10^5\, n^{2/3}\, AD^{1/2}\, C\, v^{1/2} \qquad \text{(E1)}$$

Where i_p is the peak current (amps), n is the number of transferred electrons (usually one), A is the area of the electrode (cm^2), D is the diffusion coefficient (cm^2/s), C is the concentration (mol/cm^3), and v is the scan rate (V/s).

$$\Delta i_p = \frac{nFAD_{\circ}^{\frac{1}{2}}C_{\circ}^{*}}{(\pi t_p)^{\frac{1}{2}}}\Delta\psi_p \qquad \text{(E2)}$$

Where Δi_p is the difference in peak current (amps), n is the number of transferred electrons (usually one), F is Faraday's constant (96485 C/mol), A is the area of the electrode (cm^2), $D°$ is diffusion coefficient (cm^2/s), $C°$ is the concentration (mol/cm^3), t_p is pulse width (s), and $\Delta\psi_p$ is dimensionless peak current to which is relating the peak height of SWV to the limiting current in NPV.

$$\Delta i_p = \frac{nFAD_{\circ}^{\frac{1}{2}}C_{\circ}^{*}}{\pi^{\frac{1}{2}}(\tau - \tau^{1})}\left(\frac{(1-\sigma)}{(1+\sigma)}\right) \qquad \text{(E3)}$$

Where Δi_p is the difference in peak current(amps), n is the number of transferred electrons (usually one), F is Faraday's constant (96485 C/mol), A is the area of the electrode (cm2), $D°$ is the diffusion coefficient (cm^2/s), $C°$ is the concentration (mol/cm^3), τ` is pulse width (s), τ is the time before changing to the negative potential (s), and σ is the total pulse time (s).

$$\Delta i_p = \frac{nFAD_{\circ}^{\frac{1}{2}}C_{\circ}^{*}}{\pi^{\frac{1}{2}}(\tau - \tau`)} \qquad \text{(E4)}$$

Where Δi_p is the difference in peak current (amps), n is the number of transferred electrons (usually one), F is Faraday's constant 96485 (C/mol), A is the area of the electrode (cm^2), $D°$ is diffusion coefficient (cm^2/s), $C°$ is the bulk concentration (mol/cm^3), τ` pulse width (s), and τ the time before changing to the negative potential (s).

$$LOD = \frac{ts}{slope} \qquad \text{(E5)}$$

Where t factor = 4.303 (confidence level of 95%), s the standard deviation of the 20 ng sample.
